# Microbial Etiology and Antimicrobial Resistance in Pneumonia Among Hospitalized Patients in Kazakhstan: A Systematic Review and Single‐Arm Meta‐Analysis of Prevalence Data

**DOI:** 10.1002/hsr2.72236

**Published:** 2026-03-30

**Authors:** Radmir Sarsenov, Maxim Solomadin, Alyona Lavrinenko, Vyacheslav Beloussov, Vitaliy Strochkov, Shynggys Orkara, Nurlan Sandybayev, Sergey Yegorov

**Affiliations:** ^1^ Department of Biology, School of Sciences and Humanities Nazarbayev University Astana Kazakhstan; ^2^ International Center for Vaccinology Kazakh National Agrarian Research University (KazNARU) Almaty Kazakhstan; ^3^ Research Laboratory Karaganda Medical University Karaganda Kazakhstan; ^4^ Kazakhstan‐Japan Innovation Centre Kazakh National Agrarian Research University (KazNARU) Almaty Kazakhstan; ^5^ TreeGene Molecular Genetics Laboratory Almaty Kazakhstan

**Keywords:** antimicrobial resistance, community‐acquired pneumonia, ESKAPE pathogens, hospital‐acquired infections, pneumonia, prevalence meta‐analysis

## Abstract

**Background and Aims:**

Pneumonia is an inflammatory condition of the lower respiratory tract, commonly caused by infection and associated with substantial morbidity and healthcare utilization. The burden of pneumonia on healthcare is exacerbated by limited data on pathogenic causes and associated antimicrobial resistance (AMR). Here, we conducted a literature review and synthesized data on the microbial causes and AMR in hospitalized pneumonia patients from Kazakhstan.

**Methods:**

We searched PubMed, Medline, Embase, Web of Science, the Cochrane Central Register of Controlled Trials, eLIBRARY, and CyberLeninka for observational studies of hospitalized pneumonia patients reporting microbiologically confirmed pathogen and antimicrobial resistance data from Kazakhstan. Exclusion criteria were unclear pneumonia definitions or absence of pathogen‐specific AMR data. We assessed study quality using the Joanna Briggs Institute checklist and extracted data on microbial prevalence and resistance rates by pathogen‐antibiotic combinations. We synthesized microbial etiology and AMR patterns and performed random‐effects meta‐analysis of prevalence data.

**Results:**

Nine studies represented 1534 isolates from 1474 inpatients (2008–2022). The most prevalent pathogens were *Streptococcus pneumoniae* (17.8%), *Streptococcus* spp. (17.3%), *Haemophilus influenzae* (12.6%), and *Klebsiella pneumoniae* (10.7%). Hospital‐acquired pathogens comprised 48% of isolates. *Acinetobacter baumannii* had the highest resistance, exceeding 70% across major antibiotic classes. *Escherichia coli* had high resistance to β‐lactams (> 66%) and fluoroquinolones (63%). *Pseudomonas aeruginosa* had 60% fluoroquinolone and 27% carbapenem resistance. Fluoroquinolone resistance was widespread across multiple pathogens. *K. pneumoniae* had relatively lower resistance rates. Pathogen distribution varied by clinical setting and patient population.

**Conclusion:**

Data on the etiologic causes and AMR in pneumonia are sparse in Kazakhstan and methodologically heterogeneous. Hospital‐acquired pneumonia pathogens were highly prevalent and associated with high AMR rates. These findings underscore the need for improved pathogen surveillance and antimicrobial stewardship in Kazakhstan to address the high burden of antibiotic‐resistant and hospital‐acquired pneumonia.

## 1 Introduction

Pneumonia is a lower respiratory tract condition, most commonly infectious, characterized by inflammation of the alveoli and interstitium with clinical features including cough, fever, dyspnea, pleuritic chest pain, and hypoxemia, and radiographic evidence of consolidation or infiltrates [1]. Globally, pneumonia affects approximately 450 million people annually and ranking as the fourth leading cause of death worldwide [2]. Pneumonia incidence is several times higher in developing versus developed nations [1, 2, 3]. Despite diagnostic advances, fewer than half of hospitalized pneumonia patients receive laboratory‐confirmed diagnoses through standard clinical methods [1, 4, 5], leading to over‐prescription of broad‐spectrum antibiotics and contributing to antimicrobial resistance (AMR) development.

Pneumonia is classified as community‐acquired pneumonia (CAP), occurring outside healthcare settings, or hospital‐acquired pneumonia (HAP), developing ≥ 48 h after hospital admission [1, 6]. This distinction is clinically significant due to differences in etiology, treatment, and outcomes. CAP is typically caused by *Streptococcus pneumoniae, Haemophilus influenzae, Mycoplasma pneumoniae*, and *Legionella pneumophila*, which generally respond to standard empirical antibiotic regimens [1, 6]. In contrast, HAP is associated with healthcare interventions and predominantly caused by multidrug‐resistant Gram‐negative bacteria *Pseudomonas aeruginosa, Klebsiella pneumoniae, Acinetobacter baumannii, Enterobacter species* and Gram‐positive *Staphylococcus aureus* [1].

In Kazakhstan, a Central Asian upper‐middle‐income country, infection dynamics and AMR patterns in hospitalized pneumonia patients remain poorly characterized. High regional AMR rates, exacerbated by COVID‐19‐related increases in over‐the‐counter antibiotic sales and consumption, necessitate a consolidated evidence base to guide diagnostic testing and antibiotic stewardship [7, 8, 9]. There is also limited participation of Kazakhstan in global surveillance programs, such as the World Health Organization Global Antimicrobial Resistance Surveillance System (GLASS), which provides a standardized framework for global AMR data synthesis [10].

No systematic synthesis of the literature has been conducted on regional pneumonia etiology and AMR patterns, particularly distinguishing between community‐ versus hospital‐acquired infections. To address this, we conducted a systematic review to synthesize data on microbial etiology and AMR patterns of pneumonia requiring hospitalization in Kazakhstan. Our objectives were to identify the predominant pathogens causing pneumonia and characterize their AMR profiles to inform clinical decision‐making and public health policy.

## 2 Methods

### 2.1 Study Protocol

We followed the Preferred reporting items for systematic reviews & meta‐analysis (PRISMA) for systematic reviews guidelines for methodology, study selection, data extraction, and result reporting (Supporting Information: Appendix 2–3, Table S1). The study protocol was initially registered on the Open Science Framework (https://osf.io/x26h4) as a scoping review and was subsequently modified into a systematic review based on peer reviewers′ recommendations. All analyses were prespecified in the registered protocol.

### 2.2 Eligibility Criteria

Eligibility criteria were defined using the PICOT framework.

#### 2.2.1 Inclusion Criteria

We included observational cross‐sectional studies and case series (≥ 10 patients) of hospitalized patients with clinically diagnosed community‐ or HAP, without restrictions on age or comorbidities, conducted in Kazakhstan or reporting Kazakhstan‐specific data in any language through February 2025. Eligible studies had to report original pneumonia‐specific laboratory‐confirmed microbiological data with clear case definitions, standardized antimicrobial susceptibility testing, and outcomes including pathogen prevalence and AMR.

#### 2.2.2 Exclusion Criteria

We excluded reviews, commentaries, conference abstracts without full text, case series with < 10 patients, in vitro or animal studies, studies of nonhospitalized patients or colonization only, and those lacking pneumonia‐specific data, microbiological confirmation, adequate susceptibility testing, or clear case definitions; studies with unavailable full texts were also excluded.

### 2.3 Search Strategy

We comprehensively searched PubMed, Medline (OVID), Embase (OVID), Web of Science, CENTRAL, eLIBRARY (https://www.elibrary.ru), and CyberLeninka (https://cyberleninka.ru/) for observational studies of hospitalized pneumonia. We included studies reporting original data on bacterial pathogens and antibiotic resistance without date restrictions. Searches were conducted in March 2024 and updated in February 2025.

We included observational cross‐sectional studies reporting microbial etiology and AMR data in hospitalized pneumonia patients, without age restrictions or restrictions on underlying conditions/co‐infections. Search terms included “pneumonia,” “pathogens,” “antimicrobial resistance,” “bacterial infection,” “lower respiratory tract infection,” and “Kazakhstan” (full algorithms available at https://osf.io/x26h4). We excluded studies with unclear pneumonia definitions or lacking microbiologically confirmed pathogen/AMR data specific to pneumonia. Corresponding authors were contacted for additional data when necessary.

### 2.4 Study Selection and Screening

Study selection proceeded in three stages: duplicate removal, title and abstract screening, and full‐text review. After removing duplicates using Covidence, two authors (R.S. and M.S.) independently screened all titles and abstracts against the eligibility criteria. Studies clearly not meeting the inclusion criteria were excluded at this stage. For the remaining studies, pairs of reviewers (R.S., M.S., A.L., V.B., V.S., and S.O.) reviewed full‐text articles to determine final eligibility. Disagreements were resolved through discussion; when consensus could not be reached, a third reviewer (S.Y. or N.S.) adjudicated.

### 2.5 Risk of Bias (Quality) Assessment

We assessed study quality using the Joanna Briggs Institute Critical Appraisal Checklist for Studies Reporting Prevalence Data, which evaluates 9 criteria, including sample frame appropriateness, recruitment methods, sample size adequacy, subject/setting reporting, data analysis coverage, condition identification validity, statistical analysis, and response rates [11]. Studies were scored as “Yes,” “No,” or “Unclear” and categorized as high quality (≥ 7 “Yes”), moderate quality (4–6 “Yes”), or low quality (< 4 “Yes”).

### 2.6 Data Extraction

We used Covidence for study screening and data extraction [12]. Pairs of reviewers independently extracted data using standardized forms, with disagreements resolved by a third reviewer. We extracted microbial etiology (organism types and isolate numbers), AMR data (resistant isolates per antibiotic‐pathogen combination), microbiological methods, study years, design, regions, sample sizes, and participant characteristics. When necessary, data were extracted from figures using FIJI [13] by measuring bar lengths in pixels against percentage scales to approximate numerical values, which were rounded to whole numbers and validated by contacting corresponding authors.

### 2.7 Data Analysis

We calculated microbial prevalence as simple proportions (*X*/*N*, where *X* = isolates with detected microorganism, *N* = total isolates tested) with 95% confidence intervals (95% CI) using the Wilson score method. Data were stratified by HAP‐ versus CAP‐associated pathogens. For meta‐analysis, proportions were logit‐transformed and pooled using random‐effects models with inverse‐variance weighting via weighted least squares. Results were visualized using forest plots for prevalence data and heatmaps for AMR patterns. All analyses used *α* = 0.05 and were performed in Python (3.12.6) with Seaborn (0.13.2), SciPy (1.14.1), NumPy (2.1.1), Matplotlib (3.9.2), Pandas (2.2.3), and Statsmodels (0.14.0).

## 3 Results

### 3.1 Study Selection

**Figure 1:**
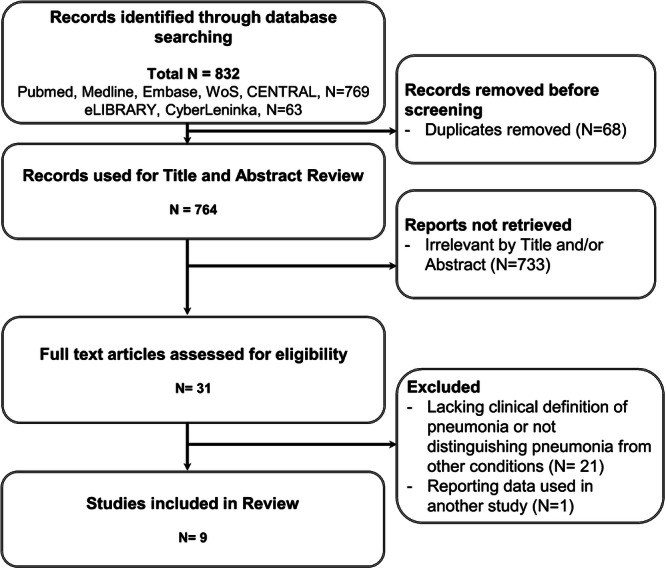
PRISMA flow diagram of the literature review process.

After removing duplicates, we identified 764 potentially relevant studies. Following title and abstract screening, 31 studies underwent full‐text review (Figure 1). We excluded studies presenting pooled data across pneumonia and other conditions [14] and included only the broadest dataset when multiple publications overlapped [15, 16]. We contacted the corresponding authors of three studies [17, 18, 19] for clarification of clinical definitions and numerical data; one author was a co‐author of this work (A.L.) [20]. One study was excluded due to author nonresponse [19] [17]. Ultimately, nine studies [16, 17, 18, 20, 21, 22, 23, 24, 25] were included (Tables 1 and 2).

**Table 1:** Characteristics of studies included in the systematic review of inpatient pneumonia etiology and antibiotic resistance in Kazakhstan.

Study	Year(s) study conducted	Reported study design	Study region(s)	Participants/isolates, *N*	Study participants	Outcomes
Studies with full texts available in English						
Ablakimova 2023 [16]	2021–2022	Retrospective, cross‐sectional analysis of medical records	Aktobe	340/340	Adult inpatients admitted with COVID‐19‐pneumonia (in 2021) or bacterial pneumonia (in 2022).	Microbial landscape and antibiotic sensitivity profiles in patients with bacterial pneumonia and/or COVID‐19
Edelstein 2013 [17]	2008–2010 (2002–2010 for entire cohort)	Cross‐sectional component for Kazakhstan. (Longitudinal for other regions).	Multinational. In Kazakhstan: Astana, Karaganda, Zhezkazgan	12/12	Adult inpatients with nosocomial *P. aeruginosa* pneumonia	Prevalence of extensively drug‐resistant MBL‐positive *P. aeruginosa*
Lavrinenko 2023 [19]	2020	Cross‐sectional, observational, multicenter	Almaty, Atyrau, Karaganda	209/281	Adult inpatients with suspected or confirmed COVID‐19 pneumonia	Bacterial coinfection and antimicrobial resistance in COVID‐19 patients
Viderman 2018 [15]	2014–2015	Retrospective observational	Astana	69/69	Adults admitted to intensive care unit (ICU), who developed ventilator‐associated pneumonia.	Etiological agents and AMR related to medical device use.
Studies with full texts available in Kazakh and/or Russian						
Bayserkeeva 2020 [20]	2019	Prospective observational	Almaty	100/100	Adult pneumonia inpatients with COPD	Etiological agents and AMR in pneumonia‐COPD inpatients
Ramazanova 2019 [21]	2014–2016	Prospective observational	Almaty	109/75	Children aged 0–5 years with radiologically confirmed pneumonia.	Bacterial etiology, prevalence of *S. pneumoniae* serotypes, and antibiotic use before hospitalization.
Sarsekeeva 2014 [22]	2013	Retrospective observational	Semey	237/236	Inpatients with CAP	Etiological agents and AMR in CAP inpatients
Usengazy 2022 [23]	2021	Retrospective observational	Aktobe	266/354	Adult inpatients with suspected or confirmed COVID‐19 pneumonia	Bacterial coinfection and antimicrobial resistance in COVID‐19 patients
Zeynebekova 2023 [24]	Unclear	Prospective observational	Karaganda	82/103	Children aged 2 months to 3 years with hospital‐admitted CAP	Clinical and bacteriological features of CAP in children with and without pneumococcal vaccination.

Abbreviations: AMR, antimicrobial resistance; CAP, community‐acquired pneumonia; COPD, chronic obstructive pulmonary disease; MBL, metallo‐β‐lactamase.

**Table 2:** Summary of microbiological assays used to identify the etiological causes and AMR in pneumonia inpatients from Kazakhstan.

Study	Microbiological assays	AMR assays	AMR interpretation guidelines
Studies with full texts available in English			
Ablakimova 2023 [16]	MicroScan AutoScan 4 (Siemens) and MicroScan Rapid (Beckman Coulter) systems.	Disk diffusion method. Internal quality control was performed using reference strains. Multidrug resistance was defined as resistance to 3 or more groups of antibiotics.	CLSI
Edelstein 2013 [17]	N/A	Agar dilution method. Imipenem—edetic acid synergy tests and MBL minimum inhibitory concentration test strips. PCR and sequencing for specific resistance genes.	EUCAST
Lavrinenko 2023 [19]	Initial characterization: Culture, morphology, and biochemistry. Species‐level identification using MALDI‐TOF MS	Disk diffusion method on Mueller‐Hinton agar, with 5% sheep blood added depending on the pathogen. Internal quality control was performed using reference strains.	CLSI
Viderman [15] 2018	“Standard microbiological methods”	AMR data unavailable. However, manuscript methods specify: Kirby‐Bauer disk‐diffusion technique.	CLSI
Studies with full texts available in Kazakh and/or Russian			
Bayserkeeva 2020[20]	Unclear	Unclear	Unclear
Ramazanova 2019 [21]	Culture, biochemical tests, PCR	N/A	N/A
Sarsekeeva 2014[22]	Unclear	Disk diffusion method	Unclear
Usengazy 2022[23]	MicroScan AutoScan 4 (Siemens)	Disk diffusion method.	CLSI
Zeynebekova 2023[24]	Initial characterization: Culture, morphology, and biochemistry. Species‐level identification using MALDI‐TOF MS	Disk diffusion method	CLSI

Abbreviations: CLSI, Clinical and Laboratory Standards Institute guidelines (CLSI M100‐24); EUCAST, European Committee on Antimicrobial Susceptibility Testing; MALDI‐TOF MS, matrix‐assisted laser desorption ionization–time of flight mass spectrometry; MBL, metallo‐β‐lactamase; PCR, polymerase chain reaction.

### 3.2 Study Characteristics

Study characteristics and microbiological methods are detailed in Tables 1 and 2. Eight studies were conducted exclusively in Kazakhstan [17, 18, 20], with one multinational study contributing cross‐sectional data on nosocomial metallo‐β‐lactamase‐positive *P. aeruginosa* from Kazakhstan [18]. Two studies focused on children [22, 25], seven on adults. Three studies included COVID‐19 patients during the pandemic [17, 20, 24], one examined chronic obstructive pulmonary disease (COPD) patients [21], and one assessed pneumonia etiology among intensive care unit (ICU) patients with various syndromes [16].

We excluded Edelstein 2013 [18] from etiology analysis as it focused solely on *P. aeruginosa*. Among the eight remaining studies, three reported CAP [20, 23, 25], one reported HAP [16], and four did not specify pneumonia source. Microbiological methods included culture‐based identification with mass spectrometry [20, 25], automated systems (MicroScan, Siemens) [17, 24], and molecular methods (polymerase chain reaction [PCR], sequencing) for bacterial and resistance gene detection [18, 22]. Four studies provided limited methodological details [16, 18, 21, 23]. Using the JBI quality assessment tool, four studies were high quality, and five were moderate quality (Table S2). Main sources of bias included inadequate sample sizes, insufficient participant characterization, and unclear microbiological and statistical methods.

**Figure 2:**
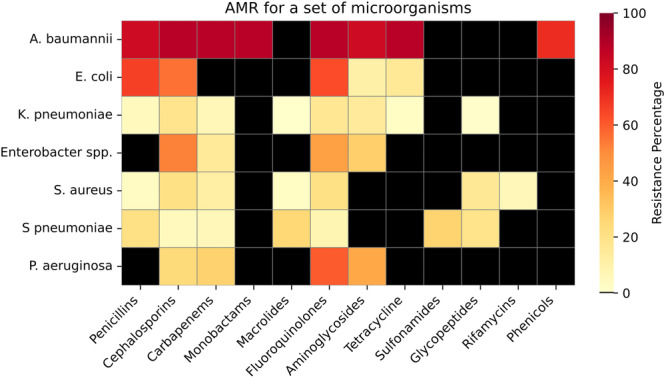
Resistance detected against 12 major antibiotic classes across 7 bacterial taxa (*Klebsiella pneumoniae*, *Staphylococcus aureus*, *Acinetobacter baumannii*, *Pseudomonas aeruginosa*, *Enterobacter* spp., *Escherichia coli*, and *Streptococcus pneumoniae*) isolated from pneumonia inpatients in Kazakhstan. Antibiotic classes represented are β‐lactams (penicillins, cephalosporins, carbapenems), aminoglycosides, fluoroquinolones, macrolides, tetracyclines, sulfonamides, and others (full list in Supporting Information: Table S5). Resistance percentages were calculated as the mean prevalence across included studies. Black squares denote a lack of available data for the corresponding pathogen–class combination. Quantitative data are given in Supporting Information: Table S5.

**Figure 3:**
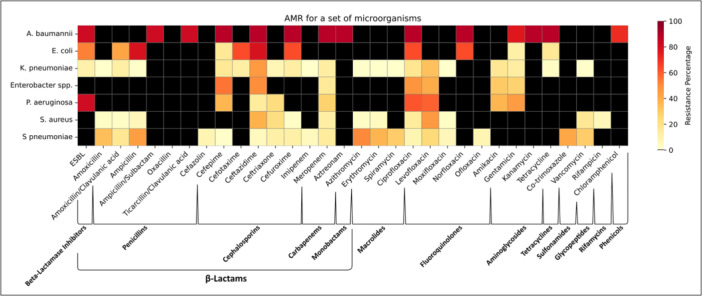
Resistance detected against 32 individual antibiotics across seven bacterial taxa isolated from pneumonia inpatients in Kazakhstan. Antibiotics include representatives of β‐lactams, aminoglycosides, fluoroquinolones, macrolides, tetracyclines, sulfonamides, and glycopeptides (full list in Supporting Information: Table S5). Bacterial taxa include *Klebsiella pneumoniae*, *Staphylococcus aureus*, *Acinetobacter baumannii*, *Pseudomonas aeruginosa*, *Enterobacter* spp., *Escherichia coli*, and *Streptococcus pneumoniae*. Resistance percentages were averaged across all available studies. Black squares indicate missing data. ESBL, extended‐spectrum β‐lactamase. Quantitative data are provided in Supporting Information: Table S5.

We did a quantitative synthesis of etiological data from eight studies (Table 3 and Supporting Information: Appendix, Table S3) and AMR data from seven studies, and analyzed nosocomial MBL‐positive *P. aeruginosa* data from one study [18] (Figures 2 and 3 and Supporting Information: Appendix Tables S4,S5).

**Table 3:** Distribution of bacterial and fungal taxa isolated from patients hospitalized with pneumonia in Kazakhstan. Total number of tested isolates = 1534. The pooled prevalence for each species was calculated using a random‐effects model on the logit‐transformed prevalence values. The weighted least squares model was then fit with inverse‐variance weighting, and the summary effect was back‐transformed from logit to prevalence using the inverse logit function. Similarly, 95% CI were back‐transformed from the logit scale. Supporting Information: Table S3 contains per‐study *X* and *N* values for Table 3 .

	Studies, *N*	Pooled prevalence, % [95% CI]
Bacteria typically associated with hospital‐acquired pneumonia		
*Klebsiella pneumoniae*	8	10.68 [7.79, 14.47]
*Staphylococcus spp*.^a^	5	8.85 [5.68, 13.53]
*Enterobacter spp*.	5	8.37 [3.46, 18.85]
*Staphylococcus aureus*	8	7.51 [4.86, 11.44]
*Acinetobacter baumannii*	2	6.64 [4.45, 9.8]
*Escherichia coli*	3	6.08 [4.2, 8.73]
*Pseudomonas aeruginosa*	7	4.47 [1.67, 11.39]
*Klebsiella oxytoca*	3	4.13 [2.94, 5.79]
*Stenotrophomonas maltophilia*	1	1.07 [0.34, 3.26]
*Citrobacter freundii*	1	0.29 [0.04, 2.06]
Bacteria typically associated with community‐acquired pneumonia		
*Streptococcus pneumoniae*	5	17.8 [5.43, 44.96]
*Streptococcus spp*.^b^	2	17.29 [7.73, 34.29]
*Haemophilus influenzae*	4	12.62 [7.47, 20.53]
*Mycoplasma pneumoniae*	1	5.83 [2.64, 12.36]
*Moraxella catarrhalis*	1	1.76 [0.79, 3.87]
Bacteria uncommon in pneumonia		
*Enterococcus faecalis*	2	5.55 [2.91, 10.33]
*Citrobacter diversus*	3	5.46 [3.72, 7.96]
*Klebsiella spp*.^c^	2	4.64 [3.3, 6.48]
*Proteus spp*.	2	3.31 [2.21, 4.94]
*Pseudomonas cepacia*	1	0.59 [0.15, 2.32]
*Bacillus spp*.	1	0.29 [0.04, 2.06]
*Agrobacterium radiobacter*	1	0.29 [0.07, 1.14]
*Vibrio spp*.	1	0.29 [0.07, 1.14]
Fungi		
*Candida spp*.^d^	5	13.13 [5.97, 26.46]

a Other than *S. aureus*: *S. epidermidis*, *S. haemolyticus*, *S. intermedius*.

^b^ Other than *S. pneumoniae*.

^c^ Other than *K. pneumoniae* and *K. oxytoca.*

^d^ Includes *C. albicans*, *C. crusei*, *C. glabrata*, *C. kefyr*, and *C. tropicalis*.

### 3.3 Primary Outcomes

#### 3.3.1 Microbial Etiology of Pneumonia

**Figure 4:**
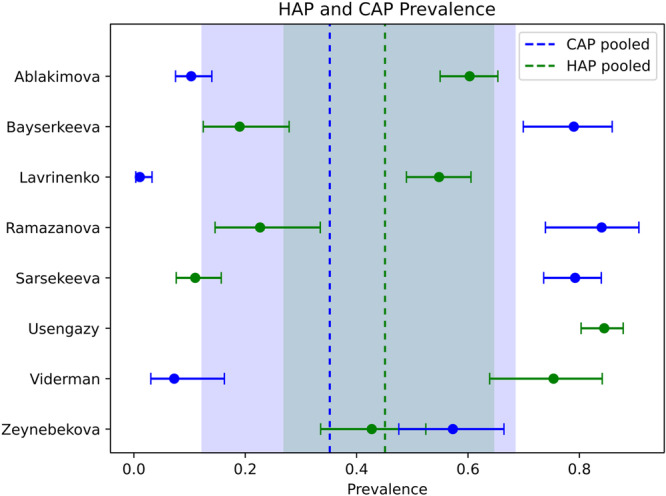
Meta‐analysis of community‐acquired pneumonia (CAP) versus hospital‐acquired pneumonia (HAP) prevalence in studies from Kazakhstan. Each circle represents prevalence from an individual study (blue = CAP; green = HAP), with horizontal bars showing 95% confidence intervals derived from logit‐transformed proportions. Pooled prevalence estimates are shown by vertical dashed lines, with shaded bands representing 95% CI. Quantitative data are given in Supporting Information: Table S4.

Across 9 studies, 1534 microbiological isolates were available from 1474 inpatients (Table 3). The most prevalent bacterial pathogens were *S. pneumoniae* (17.8%), *Streptococcus* spp. (17.3%), *H. influenzae* (12.6%), and *K. pneumoniae* (10.68%). Nearly half of all isolates (735/1534, 47.91%) were bacteria typically associated with HAP, partly reflecting study design bias as some authors specifically focused on HAP cases (e.g., Viderman and Usengazy, Figure 4). Fungi (*Candida* spp.) comprised 13.1% of isolates, representing the main nonbacterial pathogens identified.

#### 3.3.2 AMR Patterns in Pneumonia

AMR data were available for seven bacterial taxa, including five ESKAPE [26] bacteria (*S. aureus, K. pneumoniae, A. baumannii, P. aeruginosa,* and *Enterobacter spp.), E. coli,* and *S. pneumoniae* across 32 antimicrobials representing 12 classes (Figures 2 and 3, Supporting Information: Table S5).

Most data were available for the ESKAPE pathogens. *A. baumannii* had resistance rates exceeding 70% across eight major antibiotic classes. *K. pneumoniae* had relatively low resistance, with the highest rates to cephalosporins (18%) and fluoroquinolones (17%). *Enterobacter* spp. showed resistance rates of 53% to cephalosporins and 44% to fluoroquinolones. *P. aeruginosa* had resistance of 27% to carbapenems and 60% to fluoroquinolones. *S. aureus* had low resistance overall, with the highest rates of 21% to cephalosporins and fluoroquinolones.

Among the non‐ESKAPE bacteria, *E. coli* had resistance rates of > 66% to penicillins and 63% to fluoroquinolones, with lower rates to aminoglycosides (11%) and tetracyclines (16%). *S. pneumoniae* exhibited resistance rates of 32% to penicillins, 39% to macrolides, and 42% to sulfonamides.

Overall, resistance was highest in *A. baumannii* and *E. coli*, moderate in *Enterobacter* spp. and *P. aeruginosa*, and comparatively low in *K. pneumoniae, S. aureus*, and *S. pneumoniae*.

### 3.4 Secondary Outcomes

#### 3.4.1 Community‐Acquired Versus Nosocomial Pathogen Distribution

Meta‐analysis of CAP versus HAP‐associated pathogen prevalence showed substantial interstudy variation (Figure 4, Supporting Information: Table S4). Several studies [16, 17, 20, 24] predominantly identified HAP‐associated pathogens, while others [21, 22] were consistent with more typical global HAP rates (20%–25%). Zeynebekova et al. [25] reported nearly even CAP versus HAP pathogen distribution (45% vs. 55%). Notably, Sarsekeeva et al.'s CAP‐focused study [23] still reported 15% of HAP‐associated isolates, suggesting potential misclassification of CAP versus HAP conditions or community transmission of nosocomial pathogens. Taken together, the high observed prevalence of HAP‐associated organisms across studies may indicate inadequate infection control in hospitals in Kazakhstan, and/or possible spill‐over of hospital‐acquired pathogens into communities.

#### 3.4.2 Pneumonia Etiology by Clinical Setting

Pathogen distribution varied by clinical setting and patient characteristics. The ICU study [16] had Gram‐negative predominance (*A. baumannii, K. pneumoniae, P. aeruginosa*). Pediatric studies [22, 25] had higher CAP pathogen proportions (*S. pneumoniae, H. influenzae*). COVID‐19 studies [17, 20, 24] consistently showed elevated opportunistic pathogens (*Enterobacter spp., Candida spp*.). The COPD study [21] had substantial *K. pneumoniae* and *P. aeruginosa* representation.

#### 3.4.3 Geographic Distribution

Studies represented Central [16, 18, 20, 21, 22, 25], Eastern [23], and Western [17, 20, 24] Kazakhstan regions. Northern Kazakhstan was unrepresented. Geographic subgroup analysis was not feasible because most studies lacked geographic stratification, multicenter studies reported combined results, and substantial methodological heterogeneity would confound regional comparisons.

## 4 Discussion

We conducted a systematic review of pneumonia etiology and AMR in Kazakhstan. Our analysis summarizes the prevalence of microbial causes of pneumonia, attributing a substantial proportion of pneumonia acquisition to hospital settings. We observed AMR trends consistent with other recent surveys, both local [7, 8, 27] and more global [3, 9, 28]. *A. baumannii* was particularly multidrug resistant, consistent with its global designation as a critical priority pathogen [26]. Fluoroquinolone resistance was widespread across Gram‐negative isolates and present in *S. aureus*. The relatively low carbapenem resistance observed in *K. pneumoniae* suggests carbapenems may remain effective, although ongoing surveillance is essential given the potential for rapid emergence of resistance mechanisms [3].

Despite only one study explicitly focusing on HAP in our etiology analysis, the observed high prevalence of Gram‐negative bacteria (*A. baumannii, P. aeruginosa, K. pneumoniae, Enterobacter* spp.) and fungi among pneumonia inpatients suggests a substantial, and under‐reported, burden of hospital‐acquired infection in Kazakhstan. Notably, *A. baumannii, K. pneumoniae, P. aeruginosa*, and *Enterobacter* spp. along with *Enterococcus faecium* and *S. aureus* constitute the ESKAPE pathogens, leading causes of hospital‐acquired infections that often exhibit multidrug resistance [29]. Therefore, the high AMR rates we observed for pathogens such as *A. baumannii* are not surprising and are consistent with global trends showing rapidly escalating multidrug resistance among ESKAPE pathogens [29].

The substantial prevalence of *Enterobacter* spp. and fungi among pneumonia isolates was somewhat unexpected. This finding may be a result of different factors, for example, the inclusion of COVID‐19 patients in three studies [17, 20, 24]—as ~ 20% of COVID‐19 admissions are associated with various bacterial co‐infections [30]—and/or methodological factors such as sampling techniques or specimen processing associated with sample contamination. Interestingly, one of the pandemic studies collected samples within 48 h of admission [20], consistent with community‐acquired infection timing. However, the bacterial co‐infection rates in this study at least by two‐fold exceeded those observed in COVID‐19 patients in other studies [30, 31]. This discrepancy may reflect inconsistent definitions of hospital admission timing versus sample collection in the study from Kazakhstan. These observations also highlight the burden of bacterial co‐infection as another underrecognized factor in the clinical management of pneumonia in Kazakhstan. Managing co‐infections is challenging due to overlapping clinical features and compounding AMR, as multidrug‐resistant pathogens complicate treatment and increase hospitalization duration and morbidity [32].

Several bacterial pathogens associated with pneumonia were not reported by the studies from Kazakhstan. The prominent examples are *H. influenzae*, *L. pneumophila*, *M. pneumoniae, Chlamydophila pneumoniae* [1], and *Mycobacterium tuberculosis‐* highly prevalent in Kazakhstan [33]. This lack of anticipated etiological agents is likely due to a combination of factors, such as geographic specifics, seasonality, and a lack of specific tests. For example, *L. pneumophila* detection requires specialized techniques‐ such as urinary antigen tests‐ which are not routinely implemented in Kazakhstan. Similarly, *M. tuberculosis* requires specialized assays, which are not performed in standard pneumonia workups. On the other hand, we noted that in a pediatric study [22], a third of cases had Viridans streptococci (VS), rarely seen as the primary cause of pneumonia due to VS′s commensal presence in the oropharynx. However, other studies have reported associations of VS with pneumonia, calling for more attention to the role of VS in pneumonia pathogenesis [34].

Our work highlights major evidence gaps commonly seen in low‐ and middle‐income countries [9, 28, 35]. Critical subpopulations‐ including older adults, immunocompromised individuals, patients with chronic diseases, and pregnant women‐ are unstudied. Only two pediatric studies and one COPD study were identified. No studies stratified pathogen prevalence by pneumonia severity or assessed temporal or seasonal patterns. Regional coverage was limited, with no data from Northern Kazakhstan and minimal studies from Eastern and Western regions. Most studies lacked geographic disaggregation, used heterogeneous methods, or provided insufficient detail for subgroup analyses.

Several pathogens identified in our review warrant enhanced surveillance given concerning resistance profiles, such as *A. baumannii* (> 70% MDR). *K. pneumoniae*, while currently showing low carbapenem resistance, requires proactive surveillance given its propensity for rapid resistance acquisition globally [36]. Widespread fluoroquinolone resistance (60% in *P. aeruginosa*, 63% in *E. coli*, 44% in *Enterobacter* spp.) suggests declining effectiveness. Among CAP pathogens, macrolide (39%) and penicillin (32%) resistance in *S. pneumoniae* indicates a need to revise empirical treatment recommendations. Carbapenem resistance in *P. aeruginosa* (27%) and the emergence of metallo‐β‐lactamase producers also demand close monitoring.

Addressing the rapid rise of AMR across pathogens requires comprehensive public health strategies that integrate direct and indirect measures, coordinated with strong antimicrobial stewardship, infection prevention and control, and robust surveillance systems. Thus, national health systems should incorporate newer generation antibiotics against the most problematic ESKAPE pathogens, including, for example, cefiderocol and sulbactam‐durlobactam for extensively drug‐resistant *A. baumannii* [37, 38], ceftazidime‐avibactam and meropenem‐vaborbactam for carbapenemase‐producing Enterobacterales [39], ceftolozane‐tazobactam for multidrug‐resistant *P. aeruginosa* [40]. Another complementary prevention strategy is to ensure uptake of vaccines that protect against pneumonia‐causing pathogens‐ such as pneumococcal, *H. influenzae* type b, and pertussis—which are already included in the national immunization schedule. Notably, influenza vaccination provides indirect benefits against secondary bacterial pneumonia development [41, 42], although there are no published data on influenza vaccination rates, which are likely suboptimal in Kazakhstan. The high prevalence of HAP‐associated pathogens‐ considerably higher than global estimates of 10%–30%—suggests systemic infection control deficiencies requiring urgent action. Facilities should strengthen hand hygiene, environmental cleaning, MDR organism isolation procedures, and nosocomial infection surveillance with transparent reporting; external infection control audits may be warranted [28, 35].

Our analysis is limited by the small number of available studies, moderate quality ratings, and incomplete geographical coverage within Kazakhstan. Despite comprehensive searches of global and Kazakhstan‐specific databases, we may have missed nonindexed studies from the region. There were limited data on prior antibiotic use, which may have influenced pathogen isolation rates. For example, in one pediatric study, 36% of patients received preadmission antibiotics, including cephalosporins (22%), penicillins (19%), macrolides (5%), and multiple antibiotics (6%)‐ likely contributing to one‐third of samples showing no culture growth in this study [22]. Only two studies used molecular methods [18, 22], indicating predominant reliance on traditional culture. Molecular assays could improve detection accuracy, reduce antibiotic‐related false negatives, accelerate diagnostic turnaround times, and provide additional information on nonbacterial infections [43, 44, 45]. Notably, we were unable to conduct geographic subgroup analysis as most studies reported only aggregate data, several were multicenter with combined results, and only 1–5 studies were available per region, with substantial variation in methods, clinical settings, and focus. As a result, any geographic comparison would likely reflect study‐level differences rather than true regional epidemiology. Future research should prioritize standardized, nationwide surveillance using consistent microbiological methods, case definitions, and reporting frameworks aligned with both regional and international guidelines [9, 10].

## 5 Conclusions

Our study is the first to synthesize data on the etiological causes and AMR patterns in pneumonia in Kazakhstan, adding to the growing knowledge about infectious disease epidemiology in the region [46, 47, 48, 49, 50]. The unexpectedly high prevalence of hospital‐acquired infection and AMR in Kazakhstan warrants implementation of stricter hospital infection control programs, more effective national surveillance and antimicrobial stewardship, and more active involvement in global initiatives. Future studies should apply standardized case definitions aligned with international guidelines; improve detection of atypical and fastidious organisms; better distinguish CAP from HAP; document prior antibiotic exposure; and provide sufficient data granularity for risk‐stratified and geographic analyses.

## Author Contributions

**Radmir Sarsenov:** conceptualization, data curation, formal analysis, methodology, visualization, writing – original draft, writing – review and editing. **Maxim Solomadin:** conceptualization, data curation, writing – review and editing. **Alyona Lavrinenko:** conceptualization, data curation; formal analysis, writing – review and editing. **Vyacheslav Beloussov:** data curation, writing – review and editing. **Vitaliy Strochkov:** data curation, writing – review and editing. **Shynggys Orkara:** data curation, writing – review and editing. **Nurlan Sandybayev:** conceptualization, resources, writing – review and editing, project administration. **Sergey Yegorov:** conceptualization, data curation, formal analysis, methodology, visualization, writing – original draft, writing – review and editing.

## Ethics Statement

This systematic review adhered to the preferred reporting items for systematic reviews and meta‐analysis (PRISMA) guidelines. Ethical approval and informed consent were not required as this study used previously published data.

## Conflicts of Interest

The authors declare no conflicts of interest.

## Transparency Statement

The lead author Radmir Sarsenov, Nurlan Sandybayev, and Sergey Yegorov affirms that this manuscript is an honest, accurate, and transparent account of the study being reported; that no important aspects of the study have been omitted; and that any discrepancies from the study as planned (and, if relevant, registered) have been explained.

## Supporting information


**Table S1:** Preferred Reporting Items for Systematic Reviews & Meta‐Analysis (PRISMA) for Scoping Reviews guidelines **Table S2:** Results of the study quality appraisal using the Joanna Briggs Institute (JBI) quality appraisal checklist.

## Data Availability

All authors have read and approved the final version of the manuscript. N.S., S.Y., and R.S. had full access to all of the data in this study and take complete responsibility for the integrity of the data and the accuracy of the data analysis. All the data used in the study are included in the manuscript and supporting files. All scripts that we used for calculations are available on GitHub (https://github.com/mirakklys/AMR_pneumonia repository, files “AMR_heatmap.py, MA_pooled_prev_Forest_Plot.py, Single‐arm_Meta‐Analysis.py).
