# Supplementary material for: Microbial Etiology and Antimicrobial Resistance in Pneumonia Among Hospitalized Patients in Kazakhstan: A Systematic Review and Single‐Arm Meta‐Analysis of Prevalence Data
Source: Health Sci Rep. 2026 Mar 30;9(4):e72236. doi: 10.1002/hsr2.72236 (PMC13087514; doi:10.1002/hsr2.72236)
Supplement: Supplementary file 1 — Table S1: Preferred Reporting Items for Systematic Reviews & Meta‐Analysis (PRISMA) for Scoping Reviews guidelines Table S2: Results of the study quality appraisal using the Joanna Briggs Institute (JBI) quality appraisal checklist. [file HSR2-9-e72236-s001.pdf]

Microbial Etiology and Antimicrobial Resistance in Pneumonia Among Hospitalized Patients in Kazakhstan: A Systematic Review and Single-Arm Meta-analysis of Prevalence Data.

Radmir Sarsenov [1*], Maxim Solomadin [2], Alyona Lavrinenko [3], Vyacheslav Beloussov [4,5], Vitaliy Strochkov [4], Shynggys Orkara [4], Nurlan Sandybayev [4*] and Sergey Yegorov [1*]

Appendix

Table S1. Preferred Reporting Items for Systematic reviews and Meta-Analyses (PRISMA) Checklist.

Section and Topic	Item #	Checklist item	Location where item is reported
**TITLE**			
Title	1	Identify the report as a systematic review.	P1
**ABSTRACT**			
Abstract	2	See the PRISMA 2020 for Abstracts checklist.	P2
**INTRODUCTION**			
Rationale	3	Describe the rationale for the review in the context of existing knowledge.	P3
Objectives	4	Provide an explicit statement of the objective(s) or question(s) the review addresses.	P3
**METHODS**			
Eligibility criteria	5	Specify the inclusion and exclusion criteria for the review and how studies were grouped for the syntheses.	P4
Information sources	6	Specify all databases, registers, websites, organisations, reference lists and other sources searched or consulted to identify studies. Specify the date when each source was last searched or consulted.	P4
Search strategy	7	Present the full search strategies for all databases, registers and websites, including any filters and limits used.	P4 and https://osf.io/x26h4
Selection process	8	Specify the methods used to decide whether a study met the inclusion criteria of the review, including how many reviewers screened each record and each report retrieved, whether they worked independently, and if applicable, details of automation tools used in the process.	P4-P5
Data collection process	9	Specify the methods used to collect data from reports, including how many reviewers collected data from each report, whether they worked independently, any processes for obtaining or confirming data from study investigators, and if applicable, details of automation tools used in the process.	P4-P5
Data items	10a	List and define all outcomes for which data were sought. Specify whether all results that were compatible with each outcome domain in each study were sought (e.g. for all measures, time points, analyses), and if not, the methods used to decide which results to collect.	P5
	10b	List and define all other variables for which data were sought (e.g. participant and intervention characteristics, funding sources). Describe any assumptions made about any missing or unclear information.	P5
Study risk of bias assessment	11	Specify the methods used to assess risk of bias in the included studies, including details of the tool(s) used, how many reviewers assessed each study and whether they worked independently, and if applicable, details of automation tools used in the process.	P4
Effect measures	12	Specify for each outcome the effect measure(s) (e.g. risk ratio, mean difference) used in the synthesis or presentation of results.	P5
Synthesis methods	13a	Describe the processes used to decide which studies were eligible for each synthesis (e.g. tabulating the study intervention characteristics and comparing against the planned groups for each synthesis (item #5)).	P5
	13b	Describe any methods required to prepare the data for presentation or synthesis, such as handling of missing summary statistics, or data conversions.	P5
	13c	Describe any methods used to tabulate or visually display results of individual studies and syntheses.	P5
	13d	Describe any methods used to synthesize results and provide a rationale for the choice(s). If meta-analysis was performed, describe the model(s), method(s) to identify the presence and extent of statistical heterogeneity, and software package(s) used.	P5
	13e	Describe any methods used to explore possible causes of heterogeneity among study results (e.g. subgroup analysis, meta-regression).	P5
	13f	Describe any sensitivity analyses conducted to assess robustness of the synthesized results.	P5
Reporting bias assessment	14	Describe any methods used to assess risk of bias due to missing results in a synthesis (arising from reporting biases).	P4
Certainty	15	Describe any methods used to assess certainty (or confidence) in the body of evidence for an outcome.	P4

Section and Topic	Item #	Checklist item	Location where item is reported
assessment			
**RESULTS**			
Study selection	16a	Describe the results of the search and selection process, from the number of records identified in the search to the number of studies included in the review, ideally using a flow diagram.	P5
	16b	Cite studies that might appear to meet the inclusion criteria, but which were excluded, and explain why they were excluded.	P5
Study characteristics	17	Cite each included study and present its characteristics.	P5
Risk of bias in studies	18	Present assessments of risk of bias for each included study.	P6
Results of individual studies	19	For all outcomes, present, for each study: (a) summary statistics for each group (where appropriate) and (b) an effect estimate and its precision (e.g. confidence/credible interval), ideally using structured tables or plots.	PP6-7, Tables 1-2
Results of syntheses	20a	For each synthesis, briefly summarise the characteristics and risk of bias among contributing studies.	P6
	20b	Present results of all statistical syntheses conducted. If meta-analysis was done, present for each the summary estimate and its precision (e.g. confidence/credible interval) and measures of statistical heterogeneity. If comparing groups, describe the direction of the effect.	P7
	20c	Present results of all investigations of possible causes of heterogeneity among study results.	P7
	20d	Present results of all sensitivity analyses conducted to assess the robustness of the synthesized results.	P7
Reporting biases	21	Present assessments of risk of bias due to missing results (arising from reporting biases) for each synthesis assessed.	P6
Certainty of evidence	22	Present assessments of certainty (or confidence) in the body of evidence for each outcome assessed.	P7
**DISCUSSION**			
Discussion	23a	Provide a general interpretation of the results in the context of other evidence.	PP7-9
	23b	Discuss any limitations of the evidence included in the review.	P9
	23c	Discuss any limitations of the review processes used.	P9
	23d	Discuss implications of the results for practice, policy, and future research.	P9
**OTHER INFORMATION**			
Registration and protocol	24a	Provide registration information for the review, including register name and registration number, or state that the review was not registered.	P4
	24b	Indicate where the review protocol can be accessed, or state that a protocol was not prepared.	P4
	24c	Describe and explain any amendments to information provided at registration or in the protocol.	P4
Support	25	Describe sources of financial or non-financial support for the review, and the role of the funders or sponsors in the review.	P10
Competing interests	26	Declare any competing interests of review authors.	P10
Availability of data, code and other materials	27	Report which of the following are publicly available and where they can be found: template data collection forms; data extracted from included studies; data used for all analyses; analytic code; any other materials used in the review.	P10

From: Page MJ, McKenzie JE, Bossuyt PM, Boutron I, Hoffmann TC, Mulrow CD, et al. The PRISMA 2020 statement: an updated guideline for reporting systematic reviews. BMJ 2021;372:n71. doi: 10.1136/bmj.n71. This work is licensed under CC BY 4.0. To view a copy of this license, visit https://creativecommons.org/licenses/by/4.0/

Table S2. Quality Assessment of Included Studies Using the Joanna Briggs Institute (JBI) Critical Appraisal Checklist for Prevalence Studies. "Yes" were assumed to be equal to "1", "No" and "Unclear" equal to "0" in the final calculation of Quality for the study. The following scale categorized the studies quality: <4 - Low; 4-6 - Moderate; >6 - High. Studies with Moderate and High quality were selected for the analysis in our Systematic Review.

		**Studies included in the review**								
	**JBI Prevalence Checklist Questions**	**Ablakimova 2023**	**Edelstein 2013**	**Lavrinenko 2023**	**Viderman 2018**	**Bayserkeeva 2020**	**Ramazanova 2019**	**Sarsekeeva 2014**	**Usengazy 2022**	**Zeynebekova 2023**
1	Was the sample frame appropriate to address the target population?	Yes	Yes	Yes	Yes	Yes	Yes	Yes	Yes	Yes
2	Were study participants recruited in an appropriate way?	Yes	Yes	Yes	Yes	Yes	Yes	Yes	Yes	Yes
3	Was the sample size adequate?	Yes	No (n=12)	Yes	Yes	Yes	Yes	Yes	Yes	Yes
4	Were the study subjects and setting described in detail?	Yes	No (no demographic data for Kazakhstan participants)	Yes	No (no demographic data for Kazakhstan participants)	Yes	Yes	Yes	Yes	No (unclear study period)
5	Was the data analysis conducted with sufficient coverage of the identified sample?	Yes	Yes	Yes	Yes	Yes	Yes	Yes	Yes	Yes
6	Were valid methods used for the identification of the condition?	Yes	Unclear (no/limited description of microbiological methods)	Yes	Unclear (no/limited description of microbiological methods)	Unclear (no/limited description of microbiological methods)	Yes	Unclear (no/limited description of microbiological methods)	Yes	Yes
7	Was the condition measured in a standard, reliable way for all participants?	Yes	Unclear (no/limited description of microbiological methods)	Yes	Unclear (no/limited description of microbiological methods)	Unclear (no/limited description of microbiological methods)	Yes	Unclear (no/limited description of microbiological methods)	Yes	Yes
8	Was there appropriate statistical analysis?	No (lack of CI, in results/plots, inconsistencies between text and Figures)	Yes	No (lack of CI and/or other measures of statistical confidence/significance, in results/plots)	No (lack of CI and/or other measures of statistical confidence/significance, in results/plots)	No (lack of CI and/or other measures of statistical confidence/significance, in results/plots)	No (lack of CI and/or other measures of statistical confidence/significance, in results/plots)	No (lack of CI and/or other measures of statistical confidence/significance, in results/plots)	No (lack of CI, in results/plots, inconsistencies between text and Figures)	No (inconsistencies between text and Figures)
9	Was the response rate adequate, and if not, was the low response rate managed appropriately?	Unclear	Unclear	Unclear	Unclear	Unclear	Unclear	Unclear	Unclear	Unclear
	N of "Yes" responses	**7**	**4**	**7**	**4**	**5**	**7**	**5**	**7**	**6**
	Quality	**High**	**Moderate**	**High**	**Moderate**	**Moderate**	**High**	**Moderate**	**High**	**Moderate**

Table S3. Per-study prevalence (X) and total number (N) of microorganisms used for calculation of pooled prevalence for each microorganism, given in Table 3.

HAP			
**Study**	**Species**	**X (Isolates)**	**N (Tested Patients)**
Lavrinenko	Acinetobacter baumannii	17	281
Viderman	Acinetobacter baumannii	6	69
Ablakimova	Citrobacter freundii	1	340
Ablakimova	Enterobacter spp	20	340
Usengazy	Enterobacter spp	21	354
Lavrinenko	Enterobacter spp	70	281
Viderman	Enterobacter spp	9	69
Bayserkeeva	Enterobacter spp	2	100
Lavrinenko	Escherichia coli	19	281
Viderman	Escherichia coli	4	69
Zeynebekova	Escherichia coli	4	103
Ablakimova	Klebsiella oxytoca	13	340
Zeynebekova	Klebsiella oxytoca	2	103
Usengazy	Klebsiella oxytoca	17	354
Ablakimova	Klebsiella pneumoniae	60	340
Lavrinenko	Klebsiella pneumoniae	32	281
Viderman	Klebsiella pneumoniae	4	69
Sarsekeeva	Klebsiella pneumoniae	19	236
Zeynebekova	Klebsiella pneumoniae	10	103
Bayserkeeva	Klebsiella pneumoniae	8	100
Ramazanova	Klebsiella pneumoniae	1	75
Usengazy	Klebsiella pneumoniae	57	354
Ablakimova	Pseudomonas aeruginosa	14	340
Lavrinenko	Pseudomonas aeruginosa	3	281
Viderman	Pseudomonas aeruginosa	21	69
Zeynebekova	Pseudomonas aeruginosa	8	103
Bayserkeeva	Pseudomonas aeruginosa	1	100
Ramazanova	Pseudomonas aeruginosa	3	75
Usengazy	Pseudomonas aeruginosa	11	354
Ablakimova	Staphylococcus aureus	38	340
Lavrinenko	Staphylococcus aureus	10	281
Viderman	Staphylococcus aureus	4	69
Sarsekeeva	Staphylococcus aureus	7	236
Zeynebekova	Staphylococcus aureus	16	103
Bayserkeeva	Staphylococcus aureus	8	100
Ramazanova	Staphylococcus aureus	3	75
Usengazy	Staphylococcus aureus	49	354
Lavrinenko	Stenotrophomonas maltophilia	3	281
Ablakimova	Staphylococcus spp.	59	340
Ramazanova	Staphylococcus spp.	5	75
Usengazy	Staphylococcus spp.	72	354
Viderman	Staphylococcus spp.	2	69
Zeynebekova	Staphylococcus spp.	2	103
CAP			
**Study**	**Species**	**X (Isolates)**	**N (Tested Patients)**
Ablakimova	Streptococcus spp	27	340
Ramazanova	Streptococcus spp	40	75

Sarsekeeva	Streptococcus spp	40	236
Viderman	Streptococcus spp	5	69
Zeynebekova	Streptococcus spp	19	103
Ablakimova	Streptococcus pneumoniae	2	340
Lavrinenko	Streptococcus pneumoniae	3	281
Sarsekeeva	Streptococcus pneumoniae	147	236
Zeynebekova	Streptococcus pneumoniae	21	103
Bayserkeeva	Streptococcus pneumoniae	79	100
Ramazanova	Streptococcus pneumoniae	23	75
Ablakimova	Moraxella catarrhalis	6	340
Zeynebekova	Mycoplasma pneumoniae	6	103
Zeynebekova	Haemophilus influenzae	13	103
Uncommon			
Study	Species	X (Isolates)	N (Tested Patients)
Ablakimova	Citrobacter diversus	17	340
Viderman	Citrobacter diversus	1	69
Usengazy	Citrobacter diversus	23	354
Ablakimova	Agrobacterium radiobacter	1	340
Usengazy	Agrobacterium radiobacter	1	354
Viderman	Enterococcus faecalis	5	69
Bayserkeeva	Enterococcus faecalis	4	100
Viderman	Others	3	69
Sarsekeeva	Others	21	236
Ablakimova	Pseudomonas cepacia	2	340
Ablakimova	Bacillus species	1	340
Ablakimova	Klebsiella spp	14	340
Usengazy	Klebsiella spp	18	354
Ablakimova	Proteus spp	11	340
Usengazy	Proteus spp	12	354
Ablakimova	Vibrio species	1	340
Usengazy	Vibrio species	1	354
Ablakimova	Fungi	53	340
Lavrinenko	Fungi	124	281
Sarsekeeva	Fungi	2	236
Usengazy	Fungi	72	354
Viderman	Fungi	5	69

Table S4. Single-Arm etiology meta-analysis data with calculated pooled prevalence. HAP: hospital-acquired pneumonia. CAP: community-acquired pneumonia.

HAP pathogens									
Species	X	N	Prevalence	CI_Lower	CI_Upper	tau2	Q	I2(%)	95%_CI
Klebsiella pneumoniae	191	1558	10.68	7.79	14.47	0.15	26.78	73.86	10.68 [7.79, 14.47]
Staphylococcus spp.	221	1542	8.85	5.68	13.53	0.33	51.78	84.55	8.85 [5.68, 13.53]
Enterobacter spp	122	1144	8.37	3.46	18.85	1.01	70	94.29	8.37 [3.46, 18.85]
Staphylococcus aureus	135	1558	7.51	4.86	11.44	0.33	37.25	81.21	7.51 [4.86, 11.44]
Acinetobacter baumannii	23	350	6.64	4.45	9.8	0	0.62	0	6.64 [4.45, 9.80]
Escherichia coli	27	453	6.08	4.2	8.73	0	1.09	0	6.08 [4.20, 8.73]
Pseudomonas aeruginosa	61	1322	4.47	1.67	11.39	1.61	73.52	91.84	4.47 [1.67, 11.39]
Klebsiella oxytoca	32	797	4.13	2.94	5.79	0	1.67	0	4.13 [2.94, 5.79]
Stenotrophomonas maltophilia	3	281	1.07	0.34	3.26	0	0	0	1.07 [0.34, 3.26]
Citrobacter freundii	1	340	0.29	0.04	2.06	0	0	0	0.29 [0.04, 2.06]

CAP pathogens									
Species	X	N	Prevalence	CI_Lower	CI_Upper	tau2	Q	I2(%)	95%_CI
Streptococcus pneumoniae	275	1135	17.8	5.43	44.96	2.59	197.07	97.46	17.80 [5.43, 44.96]
Streptococcus spp	131	823	17.29	7.73	34.29	1.01	77.63	94.85	17.29 [7.73, 34.29]
Haemophilus influenzae	13	103	12.62	7.47	20.53	0	0	0	12.62 [7.47, 20.53]
Mycoplasma pneumoniae	6	103	5.83	2.64	12.36	0	0	0	5.83 [2.64, 12.36]
Moraxella catarrhalis	6	340	1.76	0.79	3.87	0	0	0	1.76 [0.79, 3.87]

Pathogens uncommon in pneumonia									
Species	X	N	Prevalence	CI_Lower	CI_Upper	tau2	Q	I2(%)	95%_CI
Fungi	256	1280	13.13	5.97	26.46	0.85	107.52	96.28	13.13 [5.97, 26.46]
Others	24	305	7.48	4.06	13.37	0.09	1.46	31.51	7.48 [4.06, 13.37]
Enterococcus faecalis	9	169	5.55	2.91	10.33	0	0.83	0	5.55 [2.91, 10.33]
Citrobacter diversus	41	763	5.46	3.72	7.96	0.03	2.69	25.54	5.46 [3.72, 7.96]
Klebsiella spp	32	694	4.64	3.3	6.48	0	0.37	0	4.64 [3.30, 6.48]
Proteus spp	23	694	3.31	2.21	4.94	0	0.01	0	3.31 [2.21, 4.94]

Pseudomonas cepacia	2	340	0.59	0.15	2.32	0	0	0	0.59 [0.15, 2.32]
Bacillus species	1	340	0.29	0.04	2.06	0	0	0	0.29 [0.04, 2.06]
Agrobacterium radiobacter	2	694	0.29	0.07	1.14	0	0	0	0.29 [0.07, 1.14]
Vibrio species	2	694	0.29	0.07	1.14	0	0	0	0.29 [0.07, 1.14]

Table S5. AMR data (provided as % of tested isolates) extracted from the reviewed articles.

	A. baumannii	*E. coli*	*K. pneumoniae*	*Enterobacter spp.*	*P. aeruginosa*	*S. aureus*	*S pneumoniae*
ESBL	82.35	52.63	10		81.82		
Amoxicillin			0.45			2	33.85
Amoxicillin/Clavulanic acid		42.11	11.36			2	16.92
Ampicillin		78.95	1.36			6	44.62
Ampicillin/Sulbactam	82.35						
Oxacillin							
Ticarcillin/Clavulanic acid	82.35						
Cefazolin							7.69
Cefepime	88.24	15.79	15.91	55	36.36		1.54
Cefotaxime		63.16	11.36				
Ceftazidime	88.24	78.95	45.91	50	13.64	38	4.62
Ceftriaxone			6.82		22.73	22	10.77
Cefuroxime	88.24	63.16	12.27			2	10.77
Imipenem			0.45				4.62
Meropenem	88.24		12.73	15	27.27	12	13.85
Aztreonam	88.24						
Azithromycin			0.91			2	50.77
Erythromycin			0.45			4	36.92
Spiramycin			0.45				29.23
Ciprofloxacin	88.24	63.16	17.27	47.5	61.36	14	7.69
Levofloxacin			32.73	40	59.09	46	24.62
Moxifloxacin			0.45			2	6.15
Norfloxacin	88.24	63.16					
Ofloxacin							7.69
Amikacin			16.82	30	36.36		
Gentamicin	76.47	10.53	12.73	27.5	45.45		
Kanamycin	88.24						
Tetracycline	88.24	15.79	2.73				

Co-trimoxazole							41.54
Vancomycin			0.91			16	29.23
Rifampicin						6	
Chloramphenicol	70.59						
